# Why did a Hemodialysis Patient Escape from COVID-19 Familial Cluster Infection?

**DOI:** 10.1017/dmp.2021.247

**Published:** 2021-08-04

**Authors:** Qiao Wang, Jianfeng Bao, Jiyong Huang, Lin Huang, Haichang Li, Chengping Wen

**Affiliations:** 1 School of Basic Medicine, Zhejiang Chinese Medicine University, Hangzhou, China; 2 Hangzhou Xixi Hospital affiliated to Zhejiang Chinese Medicine University, Hangzhou, China; 3 The Second Clinical Medical College, Zhejiang Chinese Medical University, Hangzhou, China

**Keywords:** SARS-CoV-2, COVID-19, familial cluster, ACE2

The globe was struck by an outbreak of Coronavirus disease 2019 (COVID-19) since late December 2019. Previous research demonstrated that COVID-19 was related to the infection of a new type of Coronavirus—Severe Acute Respiratory Syndrome-Coronavirus-2 (SARS-CoV-2).^1^ Epidemiologic statistics indicated the increased SARS-CoV-2 infectivity helped to drive the outbreak into epidemic proportions. The clinical characteristics of COVID-19 are strongly contagious, have obvious familial clustering, and middle-aged, and older males with underlying diseases are more susceptible. Familial aggregation is regarded as the dominating clinical characteristic, and familial epidemics accounted for 83% of cluster cases.^2^ Therefore, tracking and managing familial epidemics will help to effectively control the spreading infection.

Here, an unusual clinical case of a high-risk patient free of SARS-CoV-2 cluster onset has attracted our attention. Specifically, a 74-year-old man with chronic renal failure on long-term dialysis went through a clinical observation where it was found that he was frequently exposed to the household epidemic (without any defensive measures). The patient was confirmed non-COVID positive after he went through 4 Nasopharyngeal swab tests and 3 chest Computerized Tomography (CT) scans. After analyzing the uncommon case, we speculate that Angiotensin-Converting Enzyme 2 (ACE2) is the crucial ingredient to explain the difference in incidence. Based on the result of molecular modeling, ACE2 is the same virus receptor shared between SARS-CoV-2 and SARS. Furthermore, research indicated the virus attaches to host cells via the surface spike glycoproteins (S proteins) to bind to human ACE2, which may play a major role in the entry of SARS-CoV-2 into human cells.

## Low Expression Level of ACE2 in HD Patients

**Figure 1.**
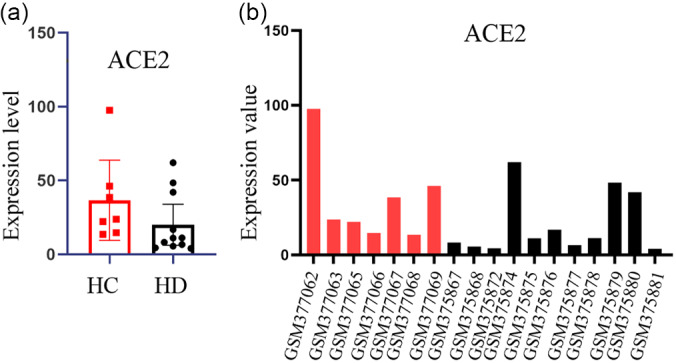
ACE2 gene expression levels in hemodialysis patients and healthy controls. Data of gene expression level of ACE2 from GSE15072 was analyzed by GEO2R online tools. (a) shows the expression level of ACE2 in hemodialysis patients and healthy controls while (b) shows the ACE2 expression value of each sample. The red column represents healthy control sample while the black represents the hemodialysis patient.

A few studies found that the expression of ACE2 decreased in patients at the end stage of hemodialysis (HD).^3^ In order to verify the results, we used online microarray data of chronic kidney disease on HD from 18 specimens for verification (11 HD, 7 health controls). Data was analyzed by GEO2R (NCBI, Maryland, USA), and we found that the expression of ACE2 on HD tends to decrease when compared to the healthy control as shown in Figure 1. The patients with hemodialysis who avoid infection may be partially explained by low level of ACE2 caused by long-term regular hemodialysis.

## Heparin treatment in HD patients derivatives block SARS-CoV-2 binding to ACE2

The patient in this case was managed with long-term low molecular weight heparin, which is the most commonly-used anticoagulant during HD procedures. A recent experiment demonstrated that it can block SARS-CoV-2 binding to ACE2 and stop its entry into host cells.^4^ Heparan Sulfate Proteoglycan (HSPG) is essential to the interaction of spike protein and ACE2 as it allows initial contact between SARS-CoV-2 and host cells on the cell surface then binds to specific input receptor-ACE2. Heparin can shift the spike structure and prevent SARS-CoV-2 invasion. Accumulated evidence has confirmed that Heparin can inhibit the enzyme activity of Heparinase, thereby inhibiting viral export and spread.^5^ Besides, the anti-inflammatory function of heparin is noteworthy, which further explains the lesser severity of most COVID infections.

Patients with end-stage renal diseases on HD are a special group that lack sufficient research. We anticipate conducting further studies to confirm the hypothesis.

## References

[1] Chen N, Zhou M, Dong X, et al. Epidemiological and clinical characteristics of 99 cases of 2019 novel coronavirus pneumonia in Wuhan, China: A descriptive study. Lancet. 2020;395(10223):507-513. 32007143 10.1016/S0140-6736(20)30211-7 PMC7135076

[2] Special Expert Group for Control of the Epidemic of Novel Coronavirus Pneumonia of the Chinese Preventive Medicine Association. An update on the epidemiological characteristics of novel coronavirus pneumonia (COVID-19). Zhonghua Liu Xing Bing Xue Za Zhi. 2020;41(2):139-144. 32057211 10.3760/cma.j.issn.0254-6450.2020.02.002

[3] Malik U, Raizada V. Some aspects of the Renin-Angiotensin-System in hemodialysis patients. Kidney Blood Press Res. 2015;40(6):614-622. 26618349 10.1159/000368537 PMC6133239

[4] Clausen TM, Sandoval DR, Spliid CB, et al. SARS-CoV-2 infection depends on cellular heparan sulfate and ACE2. Cell. 2020;183(4):1043-1057.e15. 32970989 10.1016/j.cell.2020.09.033 PMC7489987

[5] Shi C, Tingting W, Li JP, et al. Comprehensive landscape of heparin therapy for COVID-19. Carbohydr Polym. 2021;254:117232. 33357843 10.1016/j.carbpol.2020.117232 PMC7581413

